# Fluoxetine Increases the Expression of miR-572 and miR-663a in Human Neuroblastoma Cell Lines

**DOI:** 10.1371/journal.pone.0164425

**Published:** 2016-10-07

**Authors:** Mahesh Mundalil Vasu, Ayyappan Anitha, Taro Takahashi, Ismail Thanseem, Keiko Iwata, Tetsuya Asakawa, Katsuaki Suzuki

**Affiliations:** 1 Department of Psychiatry, Hamamatsu University School of Medicine, Hamamatsu, Japan; 2 Department of Neurogenetics, Institute for Communicative and Cognitive Neurosciences (ICCONS), Kerala, India; 3 Department of Child and Adolescent Psychiatry, Hamamatsu University School of Medicine, Hamamatsu, Japan; 4 Research Center for Child Mental Development, University of Fukui, Fukui, Japan; 5 Department of Biofunctional Imaging, Preeminent Medical Photonics Education and Research Center, Hamamatsu University School of Medicine, Hamamatsu, Japan; Chiba Daigaku, JAPAN

## Abstract

Evidence suggests neuroprotective effects of fluoxetine, a selective serotonin reuptake inhibitor (SSRI), on the developed neurons in the adult brain. In contrast, the drug may be deleterious to immature or undifferentiated neural cells, although the mechanism is unclear. Recent investigations have suggested that microRNAs (miRNA) may be critical for effectiveness of psychotropic drugs including SSRI. We investigated whether fluoxetine could modulate expressions of neurologically relevant miRNAs in two neuroblastoma SK-N-SH and SH-SY5Y cell lines. Initial screening results revealed that three (miR-489, miR-572 and miR-663a) and four (miR-320a, miR-489, miR-572 and miR-663a) miRNAs were up-regulated in SK-N-SH cells and SH-SY5Y cells, respectively, after 24 hours treatment of fluoxetine (1–25 μM). Cell viability was reduced according to the dose of fluoxetine. The upregulation of miR-572 and miR-663a was consistent in both the SH-SY5Y and SK-N-SH cells, confirmed by a larger scale culture condition. Our data is the first *in vitro* evidence that fluoxetine could increase the expression of miRNAs in undifferentiated neural cells, and that putative target genes of those miRNAs have been shown to be involved in fundamental neurodevelopmental processes.

## Introduction

Fluoxetine is one of the most commonly prescribed selective serotonin reuptake inhibitors (SSRIs). SSRIs can alleviate a wide variety of psychiatric symptoms such as depression, anxiety, obsession and compulsion. Underlying the clinical effects of SSRIs are multiple physiological processes, including neuroplasticity, neuroprotection, and neurogenesis in the adult brain [1]. In contrast to beneficial effects on differentiated neurons in the developed brain, SSRIs may be toxic to undifferentiated or immature cells. For instance, fluoxetine decreases the proliferation and differentiation of adipose-derived stem cells [2]. Fluoxetine also induces apoptosis in hepatic cancer cells [3], and suppresses growth of various cancer cells including lung, colon, and breast cancers, glioma and neuroblastoma [4]. With regard to the brain development, animal studies have suggested prenatal *in utero* exposure to SSRI is associated with adverse neurodevelopmental outcomes in the offspring [5, 6]. Furthermore, epidemiologic studies [7, 8] have suggested that the use of SSRIs by mother during pregnancy increases their children’s risk for autism spectrum disorder (ASD), a neurodevelopmental disorder characterized by social interaction deficits and restricted pattern of behaviours and interests [9]. These findings suggest that SSRI can affect neurodevelopment of the fetal brain, although the underlying mechanisms remain unclear.

MicroRNAs (miRNA) are small non-coding RNA molecules that post-transcriptionally regulate gene expression through sequence-specific interaction with its target messenger RNAs (mRNAs). miRNAs bind to the 3′ untranslated regions (UTR) of mRNAs to inhibit protein translation or to cause mRNA degradation [10, 11]. miRNAs have been predicted to modulate the expression of more than half of all protein coding genes[12]. Currently, 2,588 mature human miRNAs have been documented and verified (http://www.mirbase.org/cgi-bin/browse.pl?org=hsa). Approximately 50% of these miRNAs are abundantly or exclusively expressed in the brain [13], where they could be involved in fundamental processes such as the neurogenesis, neurodevelopment, and synaptic plasticity [14–19]. Concordant with these observations, aberrant miRNA expression has been detected in several neuropsychiatric disorders including ASD [20–23].

We have recently found that expressions of a group of miRNAs in the sera from children with ASD were abnormal compared with control [24]. The targets of these miRNAs were genes known to be involved in crucial neurological pathways and functions. These findings led us to assume a possible effect of SSRI on the expression of miRNAs in immature neural cells. In this *in vitro* study using undifferentiated human neuroblastoma cells, we examined whether fluoxetine could modulate the expression of miRNAs that were up- or down-regulated in children with ASD in our previous study [24].

## Materials and Methods

### Cell Culture

SK-N-SH cells (ATCC, Manassas, VA) were cultured and grown in Dulbecco's Modified Eagle's Medium (DMEM; Sigma-Aldrich, St. Louis, MD) supplemented with 10% Fetal Bovine Serum (FBS; Invitrogen, Carlsbad, CA). SH-SY5Y cells (ATCC) were maintained in 1:1 mixture of Hams F12 medium (Sigma-Aldrich) and Minimum Essential Medium (MEM, Sigma-Aldrich) supplemented with 15% FBS, 2 mM L-glutamine (Life Technologies, Tokyo, Japan) and 1% non-essential amino acids (NEAA, Sigma-Aldrich). Cells were grown at 37°C and under 5% CO_2_. Cells from the 3rd passage were used for drug experiments.

### Drug Treatment

Fifteen millimolar stock of fluoxetine hydrochloride (Sigma-Aldrich) was prepared in dimethyl sulfoxide (DMSO). Cells, seeded at a density of 5 × 10^4^ cells/well in 24 well plates, were treated with fluoxetine hydrochloride at 1-, 5-, 10-, 25-μM concentrations (3 wells each) at 50% confluency. An equal volume of DMSO was added to the control wells. The cells were harvested after 24 hours for miRNA analysis. Optimum drug concentration was chosen after assessing cell viability using trypan blue exclusion test [25].

### RNA extraction and cDNA synthesis

Total RNA, including miRNA, was extracted from harvested cells by using miRNeasy Kit (QIAGEN GmbH, Hilden, Germany) in accordance with the manufacturer’s protocol. The RNA preparation was further purified using RNeasy Mini Kit (QIAGEN). The quality and quantity of RNA were estimated using a NanoDrop 1000 Spectrophotometer (Thermo Scientific, Yokohama, Japan).

Complementary DNA (cDNA) was synthesized using the miScript II RT kit (QIAGEN). The reverse-transcription reaction mix (20 μl) was prepared using Hispec buffer for selective conversion of mature miRNAs into cDNA. The reaction mixture was incubated for 60 min at 37°C, followed by denaturation for 5 min at 95°C. Each cDNA preparation was further diluted to 220 μl with RNase-free water and stored at −20°C until use.

### Quantitative Real–Time Polymerase Chain Reaction (qPCR)

SYBR Green qPCR, performed on an ABI PRISM 7900 SDS (Applied Biosystems, Foster City, CA, USA), was used for the quantification of 13 miRNAs shown in Table 1. Ten microliters of qPCR reaction mix was prepared using the corresponding primer assays (forward primer) and a universal primer (reverse primer). All the qPCR reactions were performed in triplicate with the following cycling conditions: 95°C →15 min, followed by 40 cycles of 94°C →15 sec, 55°C →30 sec and 70°C → 30 sec. Expression of individual miRNAs was normalized against the expression of SNORD95 (QIAGEN, Accession ID: MS00033726). Fold change in gene expression between control and drug-treated cells was determined by the ΔΔCt method of relative quantification.

**Table 1:** miRNAs selected for the study with accession ID and mature sequence.

miR Name	Accession ID	Mature Sequence
hsa-miR-101-3p	MIMAT0000099	5′UACAGUACUGUGAUAACUGAA
hsa-miR-106b-5p	MIMAT0000680	5′UAAAGUGCUGACAGUGCAGAU
hsa-miR-130a-3p	MIMAT0000425	5′CAGUGCAAUGUUAAAAGGGCAU
hsa-miR-151a-3p	MIMAT0000757	5′CUAGACUGAAGCUCCUUGAGG
hsa-miR-181b-5p	MIMAT0000257	5′AACAUUCAUUGCUGUCGGUGGGU
hsa-miR-195-5p	MIMAT0000461	5′UAGCAGCACAGAAAUAUUGGC
hsa-miR-19b-3p	MIMAT0000074	5′UGUGCAAAUCCAUGCAAAACUGA
hsa-miR-320a	MIMAT0000510	5′AAAAGCUGGGUUGAGAGGGCGA
hsa-miR-328	MIMAT0000752	5′CUGGCCCUCUCUGCCCUUCCGU
hsa-miR-433	MIMAT0001627	5′AUCAUGAUGGGCUCCUCGGUGU
hsa-miR-489	MIMAT0002805	5′GUGACAUCACAUAUACGGCAGC
hsa-miR-572	MIMAT0003237	5′GUCCGCUCGGCGGUGGCCCA
hsa-miR-663a	MIMAT0003326	5′AGGCGGGGCGCCGCGGGACCGC

### Expression of SLC6A4 in SK-N-SH and SH-SY5Y

Expression of SLC6A4 protein was examined in SK-N-SH and SH-SY5Y cells was examined by Western blot analysis using anti-SERT (SAB4200039, Sigma-Aldrich, St. Louis, MO, USA) antibody [26]. The amount of total proteins in cell homogenate was measured by BCA protein assay (Rockford, Illinois, USA). Fluorescent-labelled (IRDye 800CW) anti-rabbit secondary antibody (Li-cor Biosciences, Lincoln, Nebraska) was used for the detection of protein band with an Odyssey Infrared Imaging System (Li-cor Bioscience).

qPCR was used to examine the expression of SLC6A4 mRNA in the cells. qPCR was performed in ABI PRISM 7900 Sequence Detection System by SYBR Green protocol. 16 μl reaction mixture containing 1μl cDNA (synthesized by using RT kit from Qiagen), 8 μl SYBR Green PCR Master Mix (Qiagen), 1 μl each of forward primer (5’-CTAGCAAAAGCCAGCAGGAC -3’) and reverse primer (5’-GTGACAGCCACCTTCCCTTA-3’) and 5 μl RNase free water, was used for qPCR. Reactions were performed in triplicate with the following cycling conditions: 95°C →15 min, followed by 40 cycles of 94°C →15 sec, 60°C →30 sec and 72°C → 30 sec.

### Statistical analysis

Any differential expression of miRNAs between the control and drug-treated cells was determined by Student’s *t-test*. All statistical calculations were performed using PASW Statistics 18 (IBM, Tokyo, Japan).

## Results

### Expression of SLC6A4 mRNA and the protein in the neuroblastoma cell lines

**Fig 1:**
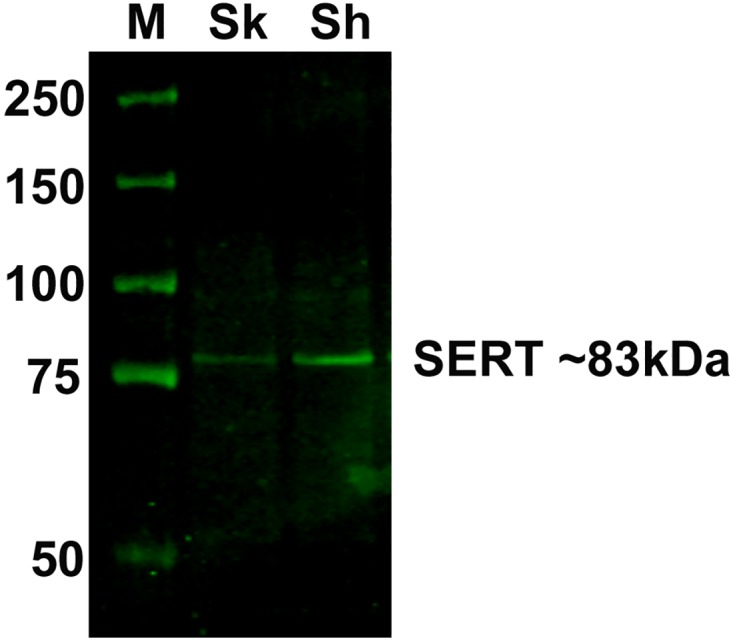
Western blot showing expression of SLC6A4 protein in SK-N-SH and SH-SY5Y cells at ~83 kDa (SAB4200039). Abbreviations: M, molecular weight marker; Sk, SK-N-SH cells; Sh, SH-SY5Y cells. Concentration of sample per lane is 1.9 ug/μl.

mRNA expression of SLC6A4 in the neuroblastoma cell lines was examined by qPCR (threshold cycle, Ct range = 30–32) and the protein expression was confirmed by Western blot (Fig 1).

### Preliminary microRNA screening

**Fig 2:**
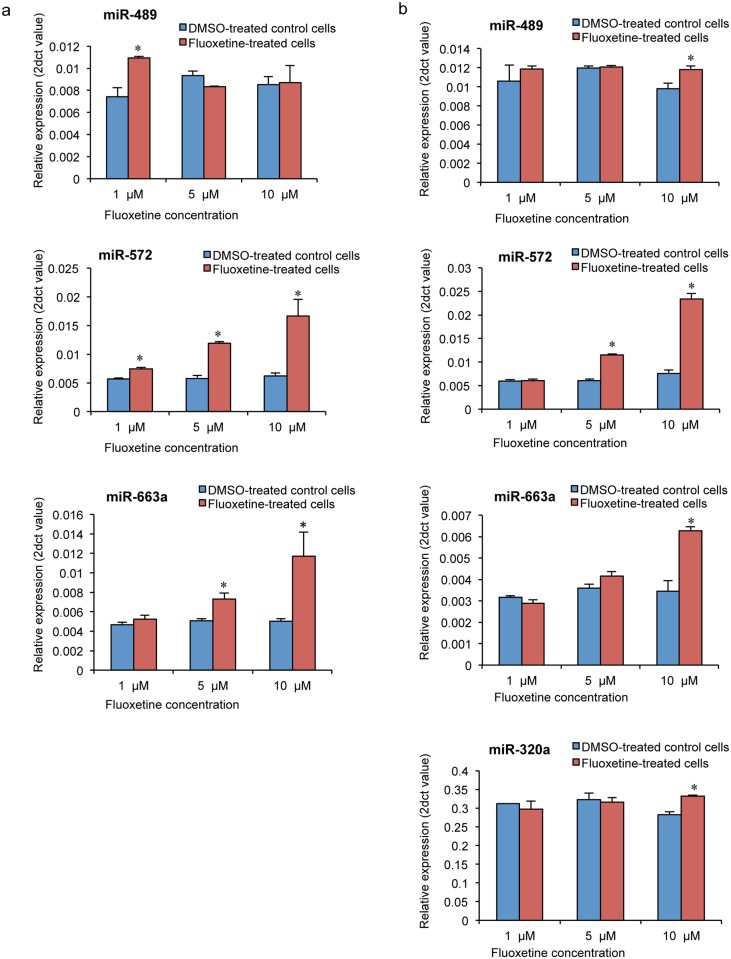
Up-regulated expression of miRNAs (miR-320a, miR-489, miR-572 and miR-663a) in (a) SK-N-SH cells and (b) SH-SY5Y cells treated with various concentrations of fluoxetine compared to DMSO-treated control cells. Error bars represents standard error. *p < 0.05.

Among the 13 miRNAs screened except miR-433, which was omitted from further analysis since the amplification was not proper in the cell lines studied, three miRNAs [miR-489 (p = 0.01 at 1μM), miR-572 (p = 0.009 at 1μM; p = 0.0004 at 5μM; p = 0.024 at 10μM); miR-663a (p = 0.032 at 5μM; p = 0.058 at 10μM)] were up-regulated in fluoxetine-treated (1-, 5- and 10-μM) SK-N-SH cells, while four miRNAs [miR-320a (p = 0.004 at 10μM), miR-489 (p = 0.037 at 10μM), miR-572 (p = 0.0002 at 5μM; p = 0.0004 at 10μM); miR-663a (p = 0.006 at 10μM)] were up-regulated in fluoxetine-treated SH-SY5Y cells compared to corresponding DMSO-treated control cells (Fig 2). Up-regulation of miRNA expression was observed at all concentrations of fluoxetine in both the cells. miR-489, miR-572 and miR-663a were consistently up-regulated in fluoxetine-treated SK-N-SH and SH-SY5Y cells. See S1 and S2 Files for supporting data.

### Cell viability

In both the cell lines, 90 ± 3% cell viability for fluoxetine was detected for concentrations 1-, 5- and 10-μM by trypan blue viability test, while at 25-μM fluoxetine the SH-SY5Y cells found 60 ± 3% cell viability but for SK-N-SH cells this was found lethal. Therefore, a concentration of 10-μM Fluoxetine was used in the subsequent validation experiments mentioned below.

### Validation

**Fig 3:**
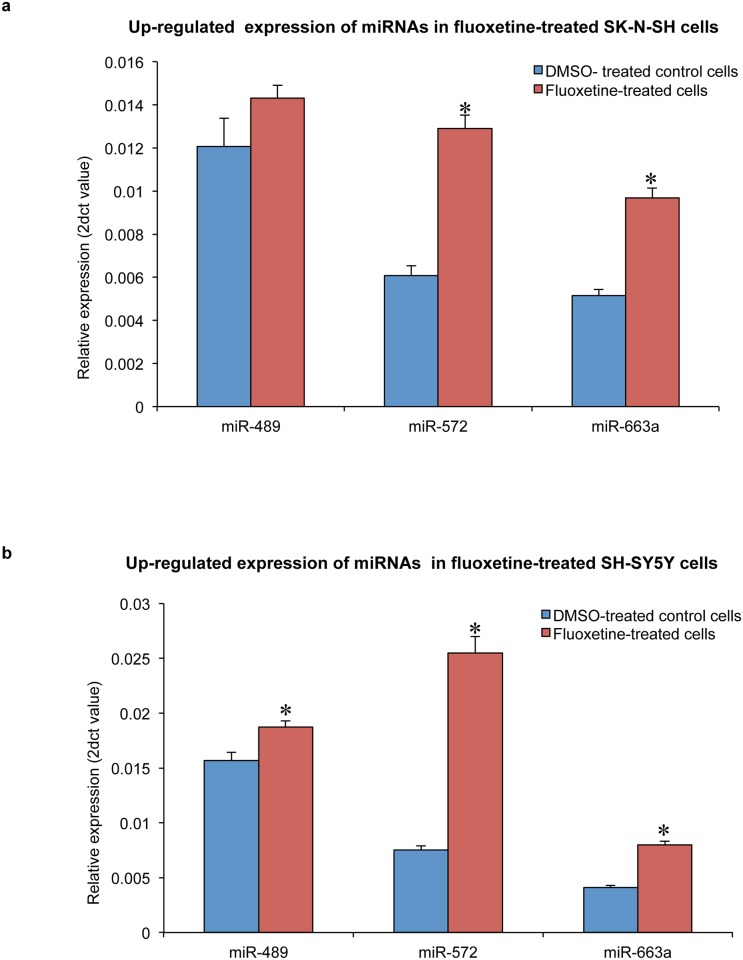
Up-regulated expression of miR-572 and miR-663a in 10 μM fluoxetine-treated (a) SK-N-SH cells and (b) SH-SY5Y cells compared to DMSO-treated control cells. Error bars represent standard error. *p < 0.05.

The expression of miR-320, miR-489, miR-572 and miR-663a were further compared between 10 μM fluoxetine (6 wells each) treated SK-N-SH and SH-SY5Y cells and their control cells. Up-regulated expression of miR-572 (ΔΔCt = 2.161, p = 4.58E-06) and miR-663a (ΔΔCt = 1.911, p = 9.75E-06) were confirmed in fluoxetine-treated SK-N-SH cells, while the up-regulated expression of miR-489 (ΔΔCt = 1.2, p = 0.007), miR-572 (ΔΔCt = 3.392, p = 3.81E-07) and miR-663a (ΔΔCt = 1.94, p = 1.77E-06) were confirmed in fluoxetine-treated SH-SY5Y cells (Fig 3). See S3 and S4 Files for supporting data.

## Discussion

The essential finding emerged from the current study was that fluoxetine (1–10 μM) up-regulated the expression of miR-572 and miR-663a consistently in two neuroblastoma cell lines SK-N-SH and SH-SY5Y. The up-regulating effect of fluoxetine was dose-dependent, and higher dose (25-μM) of fluoxetine was toxic to both cell lines. After standard therapeutic doses (40 mg per day, 30 days) administered orally, plasma levels of fluoxetine reach about 1 μM [27, 28], which is much lower than the concentration that has shown to induce upregulations of miRNAs in our study. In the brain, however, fluoxetine has ability to accumulate in the tissue due to its lipophilic property, and its concentration could reach higher levels in the brain than in plasma [29, 30]. Therefore, it is likely that the dose 5–10 μM of fluoxetine is within the clinically relevant doses. In the clinical setting, psychiatrists have used fluoxetine for treatment of patients for extended periods of time at doses higher than 100 mg per day without significant side-effects [31]. These clinical data could support our contention that SSRI fluoxetine is beneficial, but not toxic, on differentiated neurons in the developed brain.

miR-572 has been implicated in the pathogenesis of several neurological disorders such as ASD [20, 24], schizophrenia [32] and multiple sclerosis [33]. The expression of miR-572 has been found to be associated with cognitive dysfunction through down-regulation of one of its target, neuronal cell adhesion molecule 1 (NCAM1) [34]. NCAM1 is known to play an important role in neuronal cell variability, axonal proliferation, and neuronal plasticity [35, 36]. Yu et al (2015) have shown that increased miR-572 expression may lead to reduced downstream NCAM1 expression and loss of neuronal protection in patients with postoperative cognitive dysfunction [34]. Our result may be inconsistent with results by Choi et al (2012) study, in which fluoxetine regulated neuronal plasticity and neurite outgrowth by phosphorylating and activating cAMP response element-binding protein (CREB) via NCAM-induced activation of MAPK pathway, probably because they used differentiated neuron-like cells from glioma cell line. Some other target genes of miR-572 including *CDKN1A*, *DICER1*, *WNT7A* are also reported to be associated with vital neural functions, which might be down-regulated by fluoxetine-induced upregulation of miR-572 expression [37]. miR-663 has been reported as a primate-specific miRNA largely expressed in the brain [38]. miR-663a has been reported to suppress neuronal differentiation in human neural stem cells [39]. Further, it modulates the expression of genes such as *TGFβ1*, *DICER1*, *PTEN* and *VEGFA* which regulate crucial neuronal functions such as neurogenesis, neurodevelopment and synaptic plasticity [40]. In addition to miR-572 and miR-663a, the expression of miR-489 was up-regulated in fluoxetine-treated SH-SY5Y cells. miR-489, a brain specific-miRNA [41, 42], has been implicated in the pathophysiology of various neuropsychiatric disorders [43–46, 24]. Alteration of miR-489 and its target genes have been observed in postmortem brain samples from schizophrenic [43] and depressed suicidal patients [44]. The importance of miR-489 in synaptic transmission has been demonstrated in a mouse model of Alzheimer's disease [45]. In our previous study, target genes of miR-489 have been identified and predicted to be involved in important signalling systems such as *MAPK*, *ErbB*, *TGF*-beta and hedgehog signalling [24]. Nevertheless, whether the changes of miRNAs after fluoxetine exposure will lead to a significant alteration of respective targets and their biological functions requires further investigation.

The mechanism by which fluoxetine caused upregulation of miRNA expression was unknown. Since two lines of neuroblastoma cells we used in this study expressed serotonin transporter detectable at mRNA and protein levels, the simplest explanation for the upregulation of miRNA expression by fluoxetine would be binding of the drug with serotonin transporter. This is however unlikely, because the reported affinity (*Ki*) of fluoxetine for serotonin transporter is on the order of 1 nM [47], the value is far lower than the 5–10 μM concentration that we found a significant upregulation of miRNA expression. Although mechanism of antidepressant action of fluoxetine is associated with the inhibition of serotonin transporters, non-serotoninergic effects of fluoxetine have been reported. For instance, Stepulak et al. (2008) reported that fluoxetine inhibited the growth of lung and colon cancer cells as a result of inhibition of ERK1/2 activation [4]. It has also been reported that various antidepressants including fluoxetine were able to induce translocation of glucocorticoid receptor to nucleus and modulate transcription in fibroblast cells, human lymphocytes and neural cell line [48, 49]. Further study using experimental animals that examine expression levels of miRNAs in offspring from mothers having treated with SSRI during pregnancy would be warranted in this regard.

To the best of our knowledge, our data is the first *in vitro* evidence that fluoxetine could increase the expression of some miRNAs implicated in ASD. Target genes of these miRNA will be a focus of future studies, and it would be also important to validate the current findings in primary neurons and in vivo models of ASD.

## Supporting Information

S1 File Preliminary qPCR experimental data file of SH-SY5Y cells with three different concentrations (1-, 5-, 10- and 25-μM) of Fluoxetine against equal concentration of DMSO control. (XLSX)

S2 File Preliminary qPCR experimental data file of SK-N-SH cells with three different concentrations (1-, 5- and 10-μM) of Fluoxetine against equal concentration of DMSO control. (XLSX)

S3 File Confirmation qPCR experimental data with four miRNAs (miR-320, miR-489, miR-572 and miR-663a) of SH-SY5Y cells with 10-μM Fluoxetine and equal concentration of DMSO control. (XLSX)

S4 File Confirmation qPCR experimental data with four miRNAs (miR-320, miR-489, miR-572 and miR-663a) of SK-N-SH cells with 10-μM Fluoxetine and equal concentration of DMSO control. (XLSX)
